# Autoimmune gene expression profiling of fingerstick whole blood in Chronic Fatigue Syndrome

**DOI:** 10.1186/s12967-022-03682-3

**Published:** 2022-10-25

**Authors:** Zheng Wang, Michelle F. Waldman, Tara J. Basavanhally, Aviva R. Jacobs, Gonzalo Lopez, Regis Y. Perichon, Johnny J. Ma, Elyse M. Mackenzie, James B. Healy, Yixin Wang, Sarah A. Hersey

**Affiliations:** 1 Bristol Myers Squibb, Princeton, NJ 08540 USA; 2 DxTerity®, Rancho Dominguez, CA USA; 3 Rutgers Robert Wood Johnson Medical School, New Brunswick, NJ USA

**Keywords:** Chronic fatigue syndrome, Gene expression profiling, Autoimmune disease, T cell, B cell, Patient centric approach

## Abstract

**Background:**

Myalgic Encephalomyelitis/Chronic Fatigue Syndrome (ME/CFS) is a debilitating condition that can lead to severe impairment of physical, psychological, cognitive, social, and occupational functions. The cause of ME/CFS remains incompletely understood. There is no clinical diagnostic test for ME/CFS. Although many therapies have been used off-label to manage symptoms of ME/CFS, there are limited, if any, specific therapies or cure for ME/CFS. In this study, we investigated the expression of genes specific to key immune functions, and viral infection status in ME/CFS patients with an aim of identifying biomarkers for characterization and/or treatment of the disease.

**Methods:**

In 2021, one-hundred and sixty-six (166) patients diagnosed with ME/CFS and 83 healthy controls in the US participated in this study via a social media-based application (app). The patients and heathy volunteers consented to the study and provided self-collected finger-stick blood and first morning void urine samples from home. RNA from the fingerstick blood was tested using DxTerity’s 51-gene autoimmune RNA expression panel (AIP). In addition, DNA from the same fingerstick blood sample was extracted to detect viral load of 4 known ME/CFS associated viruses (HHV6, HHV7, CMV and EBV) using a real-time PCR method.

**Results:**

Among the 166 ME/CFS participants in the study, approximately half (49%) of the ME/CFS patients reported being house-bound or bedridden due to severe symptoms of the disease. From the AIP testing, ME/CFS patients with severe, bedridden conditions displayed significant increases in gene expression of IKZF2, IKZF3, HSPA8, BACH2, ABCE1 and CD3D, as compared to patients with mild to moderate disease conditions. These six aforementioned genes were further upregulated in the 22 bedridden participants who suffer not only from ME/CFS but also from other autoimmune diseases. These genes are involved in T cell, B cell and autoimmunity functions. Furthermore, IKZF3 (Aiolos) and IKZF2 (Helios), and BACH2 have been implicated in other autoimmune diseases such as systemic lupus erythematosus (SLE) and Rheumatoid Arthritis (RA). Among the 240 participants tested with the viral assays, 9 samples showed positive results (including 1 EBV positive and 8 HHV6 positives).

**Conclusions:**

Our study indicates that gene expression biomarkers may be used in identifying or differentiating subsets of ME/CFS patients having different levels of disease severity. These gene targets may also represent opportunities for new therapeutic modalities for the treatment of ME/CFS. The use of social media engaged patient recruitment and at-home sample collection represents a novel approach for conducting clinical research which saves cost, time and eliminates travel for office visits.

## Background

Chronic Fatigue Syndrome (CFS) and Myalgic Encephalomyelitis (ME) collectively known as ME/CFS, has a mean prevalence of 1.40 ± 1.57% in the worldwide general population [1]. Since 2015 there has been a rough doubling of the ME/CFS prevalence and economic impact figures in the US, with low-end prevalence assessed at 1.5 million people and economic impact in the range of 36–51 billion dollars per year [2]. These figures are comparable to losses of other chronic illnesses, suggesting that ME/CFS presents one of the highest healthcare and socioeconomic burdens [3].

The disease is defined by chronic debilitating fatigue lasting more than 6 months and various other symptoms such as unrefreshing sleep, mental and physical pain, neurological and cognitive impairment, as well as autoimmunity or immunodeficiencies [4, 5]. The onset of ME/CFS occurs most often between 30 and 50 years of age, with approximately 2–3 times more women afflicted than men [6]. The most troublesome symptoms include fatigue (85%), pain (65%), cognitive impairment (50%), orthostatic intolerance (45%), sleep disturbance (35%), post-exertional malaise (30%), and neurosensory disturbance (30%) [7]. The illness is usually relapsing and remitting in nature [8]. In the Bell study, 20% of patients continue to be ill with limitations or disability even 13 years after the onset of the syndrome [9].

The cause of the disease is unknown, and there is no specific diagnostic tool available beyond a thorough medical history and physical examination to help enable a diagnosis. The CFS diagnosis is often one of exclusion of other sources of fatigue versus a confirmation of the condition itself. Many autoimmune disease patients exhibit fatigue symptom. The prevalence of fatigue among primary Sjögren’s syndrome patients ranges between 38 and 88%, and almost 80% in multiple sclerosis (MS) [10]. Physicians report difficulty identifying CFS because the diagnostic criteria are still being adopted and many of the symptoms overlap with those of other chronic diseases. Since 1986, 25 case definitions/diagnostic criteria for CFS were created [11]. The underdiagnosis of CFS is compounded by the fact that there is a lack of disease awareness within the public.

Viral and bacterial infection are the most often linked trigger for the disease. For example, up to 75% of individuals with CFS report a viral illness as a precipitant. Influenza, common cold viruses, infectious mononucleosis, and many other viruses have been implicated [4]. More frequently, infections of cytomegalovirus (CMV), Epstein‐Barr virus (EBV), and human herpesvirus‐6 (HHV‐6) are suspected as etiological agents for ME/CFS [12]. There is a striking similarity in symptoms between long hauler COVID-19 cases and ME/CFS. An unprecedented wave of CFS-like illness related to COVID might appear over the next few years, with profound societal costs [7, 13–15].

There is convincing evidence that at least a subset of ME/CFS cases have an autoimmune etiology. Dysregulation of the immune system, autonomic nervous system and metabolic disturbances contribute to this complex syndrome, in which severe fatigue and cognitive impairment are central features [16, 17]. Immune dysregulation in ME/CFS has been frequently described including changes in cytokine profiles and immunoglobulin levels, T- and B-cell phenotype and a decrease of natural killer cell cytotoxicity [12, 18]. Autoantibodies against various antigens have been identified in ME/CFS individuals by several groups. Consistently, clinical trials such as the one from the Norway study have shown that B-cell depletion with Rituximab results in clinical benefits in about half of ME/CFS patients [19].

Currently there are no effective therapeutics for CFS. Physicians have employed the use of off-label therapies for the treatment of ME/CFS symptoms. There have been very few randomized clinical trials with investigational new drugs due to inability to accurately diagnose the disease, characterize the disease and/or sub-segment the patients [3]. In autoimmunity, two diseases with similar manifestations may have different dysregulated pathways, while diseases with very different clinical presentations often share the same immunology and genetics [20]. As a result, there have been very few therapeutic advances in the management of this debilitating disease. Recently, the link between CFS and autoimmune diseases has generated substantial interests to explore out-of-box therapeutics. The autoimmune field is accelerating its search for better biomarkers to develop novel targeted therapies [20].

Our study focused on analyses of blood samples from patients with CFS and controls by using gene expression and viral infection testing. The study enabled identification of potential biomarkers and therapeutic targets associated with CFS as well as disease severity by comparing the CFS disease group in total and subsets with the control group. Furthermore, the analysis may help understand the underlying biological mechanism implicated in the CFS disease.

## Methods

### Study design

**Fig. 1:**
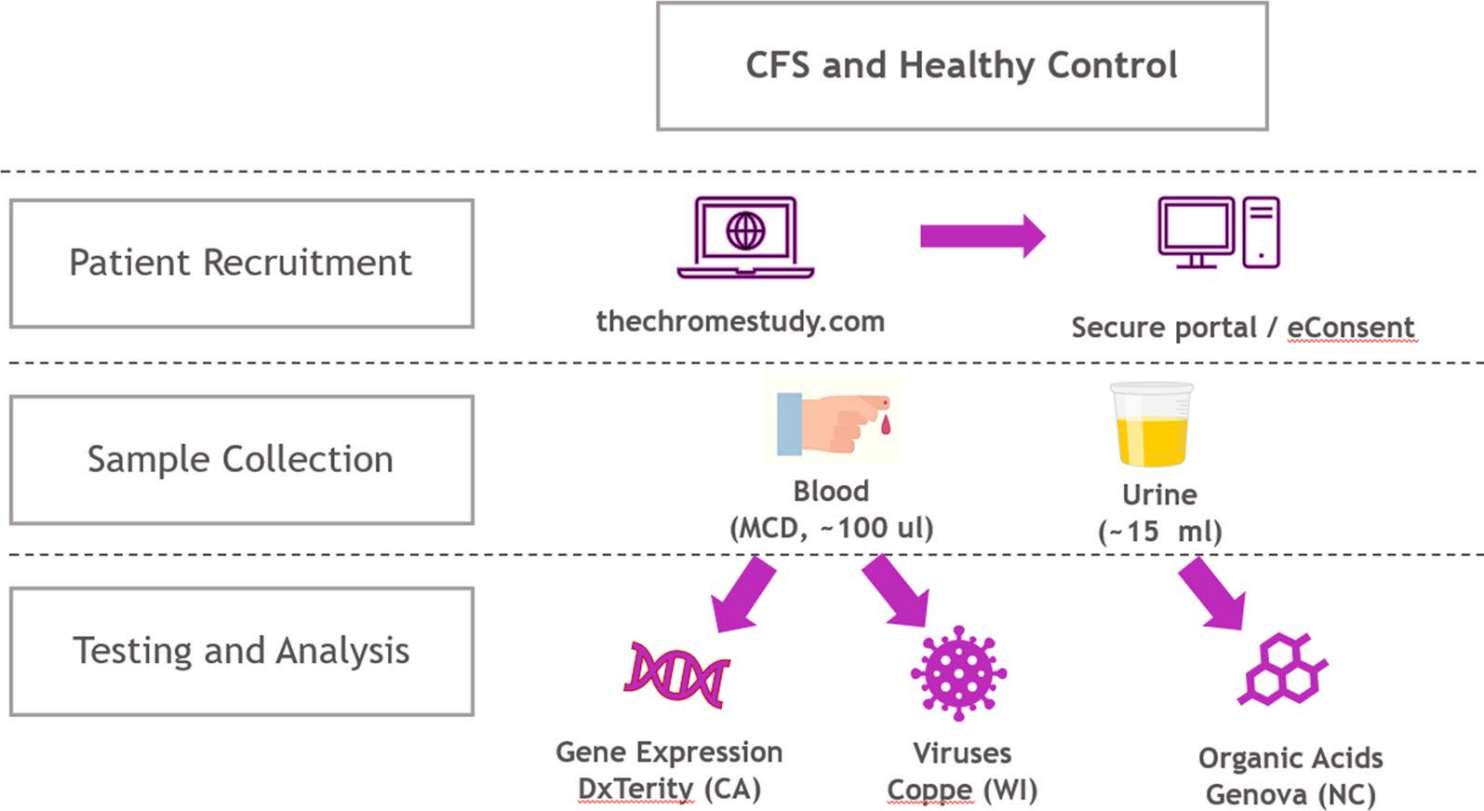
Study design and workflow

This study protocol was approved by the Western Institutional Review Board and divided into three phases: patient recruitment, sample collection and testing & analysis. Figure 1 depicts the study design and its workflow. During the patient recruitment phase, a protocol was written to allow for direct-to-participant (D2P) study implementation. The study protocol and supporting documents were submitted to a central IRB for ethical review and approval. The study originally aimed to collect samples at a single timepoint from 100 CFS patients and 100 healthy control participants, followed by samples from a second timepoint from 10% of each group to assess sampling repeatability. During the sample collection phases, subjects were recruited, qualified, consented, characterized, and sampled per the study protocol. During the testing & analysis phase, whole blood fingerstick samples were tested using the autoimmune profile (AIP) test at DxTerity (Rancho Dominguez, CA). A portion of the whole blood samples was extracted to yield DNA which was tested for viral infection using a real time PCR method at Coppe Laboratories (Waukesha, WI). The urine samples were tested for organic acid profiles at Genova Diagnostics (Asheville, North Carolina). The urine data is not the subject of this report.

### Recruitment of subjects

Upon IRB approval, participants were recruited directly through online advertising, patient advocates and advocacy groups, registries, clinical sites, and physician referral. CFS participants were able to register for the study in late May 2021 through the study specific website thechromestudy.com. Enrollment kicked off in June with invitations sent to registered participants. The website allowed potential participants to sign up and complete an initial qualification before receiving an email linking to a secure portal for further Qualification and eConsent, including identity verification. No other identifying information was stored by the study until consent was obtained. Informed consent was conducted electronically through a HIPAA compliant mobile app, where participants were able to self-report demographics and medical information. All subject-pertaining materials were reviewed by the IRB. Next, participants (male and female age 18 or older at the time of consent) completed a single study collection event consisting of one Micro Collection Device (MCD) for fingerstick blood and one first morning void urine collected from home. 20% of the ME/CFS and 19% of the control group completed a second study collection event approximately 3 to 4 weeks after the initial study collection to assess sampling repeatability. For the Disease Group, patients must have been either diagnosed with ME/CFS by their physicians, or met CFS definitions (per 2015 Institute of Medicine (IOM) diagnostic criteria for ME/CFS definition) [21] as indicated on the qualification questionnaire. For the Control Group, participants were eligible if they did not have history of fatigue and did not meet the ME/CFS definition [21].

### Self-reported data collection

Once enrolled in the study, participants were provided access to a study portal where they could access and complete the electronic patient reported outcome (ePROs) questionnaire. Participants were asked to self-report and complete ePROs about the following:

- Demographics (Age, Gender assigned at birth, Height, Weight, Race and Ethnicity)
- History and status of illness (first collection time only)
  - Year that ME/CFS symptoms started
  - Do ME/CFS symptoms result in being house bound?
  - Triggering Event (Not identifiable, bacterial infections/illness, viral infection/illness, physical trauma, emotional trauma or other)
  - Diagnosis by Health Care Professional?
  - Managed by a physician for ME/CFS condition?
  - How well are conditions/symptoms managed?
- Current medications and treatment history
  - Antibiotics
  - Chemotherapy
  - Non-steroidal anti-inflammatory drugs
  - Other anti-inflammatory drugs
  - Antihistamines
  - Immunomodulatory drugs
  - Dietary supplements
- Medical history
  - Diagnosis with autoimmune disease
  - Other conditions/diagnostics
- Involvement in another study/clinical trial for ME/CFS Study experience.

### Ethical statement

This study was approved by the Institutional Review Board and all patients gave written, informed consent.

### Sample collection

Each qualified participant received a DxCollect® MCD Fingerstick Kit and a Genova Diagnostics® Urine Collection Kit and was also asked to complete a study-related questionnaire. This was an observational study and participation did not affect any aspect of patient treatment.

The MCD blood collection kit was used to facilitate the collection, stabilization, and shipping of a microsample (about 120 μL) of blood for non-clinical use. The device was designed to collect fingerstick blood samples autonomously. The MCD kit was shipped to the subject’s home. The completed kit was returned to the lab for analysis in a pre-labeled, postage-paid box according to applicable federal regulations. The MCD sample was stable at ambient temperatures for up to 14 days.

A urine sample was collected in a 15 mL tube pre-coated with a stabilizer containing a preservative. Upon mixing after urine collection, the urine sample was frozen down at − 20 °C or less before shipment was made within 24 h of collection. Frozen urine from the participant’s home was shipped following manufacturer’s instructions for Genova Diagnostics metabolomic testing. Urine samples that did not conform to shipping and/or temperature requirement were rejected and destroyed by the lab per standard operating procedures at the time the sample was received.

### Sample analysis

#### AIP testing

The MCD blood samples were received by DxTerity where AIP gene expression testing was carried out using a method that combines an RNA-stabilizing buffer and the target-dependent chemical ligation of probes, followed by PCR amplification of the ligated probes to perform the quantitative analysis of multiple transcripts [22]. Fifty-one genes on the AIP panel were separated by capillary electrophoresis and the gene expression levels were calculated relative to the geometric mean of 3 house-keeping control genes. The results were grouped into Modules which demonstrate correlation to immune pathways or therapeutic targets.

#### Viral infection testing

DNA from MCD blood samples were extracted at DxTerity. The extracted DNA samples were shipped to an independent lab (Coppe Laboratories, Waukesha, WI) for viral infection testing for HHV6-A, HHV6-B, HHV-7, EBV and CMV. The viral load for each virus was determined via extensively validated clinical diagnostic molecular qPCR assays at Coppe Laboratories.

### Statistical data analysis

Computational data analyses were performed on the resulting data sets generated from the RNA and the DNA tests to discover potential genes associated with ME/CFS and/or sub-segmentation of ME/CFS patients based on disease severity and autoimmune disease comorbidities.

**Fig. 2:**
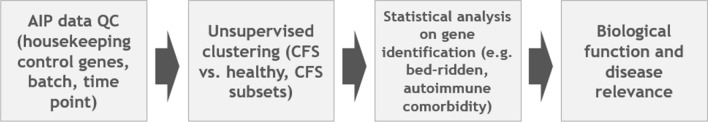
Data analysis workflow for AIP gene expression analysis

The following diagram (Fig. 2) depicts the data analysis workflow for AIP gene expression data.

#### Normalization and batch quality control

In AIP testing, results from first timepoint (n = 221) and second timepoint (n = 45) were compared to assess biological concordance between replicate samples and confirm reproducibility of the proposed normalization procedure. AIP testing was performed in two batches depending on sample availability, with batch 1 (n = 224) and batch 2 (n = 43). Each batch contained a mixture of both timepoints.

Wilcoxon signed-rank tests were performed between replicates on both the raw and normalized relative florescent units (RFU) of three housekeeping genes (ACTB, GAPDH, TFRC). Coefficient of variation of housekeeping and PCR control levels were observed to ascertain their influence on possible variations in gene expression. Additionally, raw values were re-normalized by subtracting the raw RFU of each gene from the geometric mean reference expression of each patient for comparison to the initial results obtained from the DxTerity’s AIP Testing.

#### Unsupervised clustering and obtaining genes of interest

The Bioconductor package clusterExperiment [23] was used as a framework for resampling-based sequential ensemble clustering (RSEC) on the module genes. This workflow implements several clustering iterations over a range of both standard and user-defined tuning inputs such as k0 and alpha values, dimensionality reduction methods, and cluster sizes. A single consensus clustering is determined from several candidates and further merging of related hierarchical sister nodes is then performed.

Clustering was performed on the CFS cohort to reveal potential subgroups of interest with a required minimum consensus sample size of 5. The consensus yielded 6 major groups, denoted by m01–m06 (standard RSEC naming convention). The clinical and demographic composition of each cluster was tallied and assigned a category if 80% or more of the cluster contained that category type, i.e. if 80% of the cluster was female, the cluster was labeled as majority female.

Multivariable regression models were developed from the normalized RFU of all 51 panel genes. Models controlled for demographic factors such as age, sex, and race. Clinical indications such as symptom duration, trigger event (yes/no), and whether the trigger event was based on prior viral or bacterial infection (yes/no), were also included in the additive model. Genes significantly (p < 0.05) differentially expressed by each attribute were annotated within rows of heatmaps to visually segment gene clusters of interest.

#### Heatmaps of AIP genes

Heatmaps for AIP genes were derived from log2 normalized RFU values. All heatmaps were scaled and centered prior to calculating Euclidean distances to represent dendrogram clusters.

#### Regression bootstrapping

Regression models from genes which were borderline modulated between CFS bedridden and CFS non-bedridden patients (as determined based on prior multivariable regression models) underwent residual resampling to account for sample bias and potential noise in the dataset. Coefficient confident intervals were reported from 2,000 resampling iterations.

## Results

### Patient centric enrollment and sampling: direct-to-participant (D2P)

The first participant enrollment was received on the same day the study invitation was sent out and the first sample was received just 8 days after the study invitation. The ME/CFS arm was fully enrolled within 3 months, and the control arm was fully enrolled within 4.5 months. A total of 300 participants were enrolled, with only 3 subjects having withdrawn from the study after the samples were collected. In the study, we included 267 valid AIP results, after excluding QC fails and disqualified samples. Participant questionnaire was completed by 287/297 (96.3%) of the total participants. Ten participants who did not complete the questionnaire were equally split between the CFS group and the control group. Results were compiled using a de-identified participant ID in compliance with HIPPA regulations.

### Patient characteristics

A total of 166 CFS and 83 healthy participants (controls) were recruited for the study. From the participants who completed the questionnaire, there were 125 females (75%) and 38 males (23%) in the ME/CFS group and 44 females (53%) and 34 males (41%) in the control group (Table 1). The average age was 50.8 in the ME/CFS participants and 44.8 in the controls (p = 0.001, Table 1). Average Body Mass Index (BMI) was very similar at 26.8 in the ME/CFS patients and 25.8 in the controls (p = 0.522, Table 1). Based on BMI standard weight status categories, similar percentage of the ME/CFS participants and the controls fell into underweight, healthy weight, overweight and obesity categories (Table 1) with no statistical differences (p > 0.05). Ninety-two percent (92%) of the ME/CFS participants and fifty-two (52%) of the controls were Caucasian. A much smaller percentage of both the groups were Asians, Hispanics, African Americans, and other.

**Table 1:** Cohort characteristics

	ME/CFS	Controls	Mann–Whitney U test
Total # unique subjects	166	83	*NA*
Age (years)	50.8 ± 12.8	44.8 ± 14.8	*p* = *0.00124*
Gender			
Female	125	44	NA
Male	38	34	NA
Unreported	3	5	NA
BMI (kg/m^2^)	26.8 ± 6.6	25.8 ± 5.0	*p* = *0.522*
Underweight (< 18.5)	5 (3%)	2 (2%)	NA
Healthy Weight (18.5–24.9)	72 (43%)	34 (41%)	*p* = *0.896*
Overweight (25.0–29.9)	46 (28%)	28 (34%)	*p* = *0.569*
Obesity (> 30.0)	40 (24%)	13 (16%)	*p* = *0.271*
Unreported	3 (2%)	6 (7%)	NA
Ethnicity			
Asian	2 (1%)	26 (31%)	NA
Caucasian (White)	152 (92%)	43 (52%)	NA
Hispanic (non-White)	4 (2%)	4 (5%)	NA
Other	5 (3%)	4 (5%)	NA
African American/Black	0 (0%)	1 (1%)	NA
Unreported	3 (2%)	5 (6%)	NA

Of the 166 ME/CFS participants, ninety-four percent (94%) reported receiving a diagnosis by a health care professional (Table 2). Three percent (3%) were self-assessed based on descriptions provided in the questionnaire as they considered themselves to have the symptoms listed in the 2015 IOM diagnostic criteria for ME/CFS (Table 2). The other three percent (3%) did not provide information, but they were placed in the ME/CFS group since these individuals identified as ME/CFS specific when enrolling in the study (Table 2). Similarly, out of 83 normal participants, ninety-four percent (94%) of the subjects enrolled as healthy normal controls based on the exclusion criteria (Table 2). Six percent (6%) did not provide information but were placed in the normal control group since these individuals identified as normal when enrolling (Table 2).

**Table 2:** Summary of patient medical history

	ME/CFS	Controls
Duration of CFS disease (Year)		
1–5	28 (17%)	NA
6–10	30 (18%)	NA
11–15	22 (13%)	NA
16–20	20 (12%)	NA
21–30	34 (21%)	NA
31–63	26 (16%)	NA
Unreported	4 (2%	NA
Diagnosed with ME/CFS or experiencing symptoms of ME/CFS		
Yes	161 (97%)	NA
No	NA	78 (94%)
Unreported*	5 (3%)	5 (6%)
Diagnosed with ME/CFS by a Health Care Professional		
Yes	156 (94%)	NA
No	5 (3%)	NA
Unreported*	5 (3%)	NA
Severity of ME/CFS symptoms (house-bound or bedridden)		
Yes	82 (49%)	NA
No	79 (48%)	NA
Unreported*	5 (3%)	NA
Triggering event leading to ME/CFS symptoms		
Yes	126 (76%)	NA
No	35 (21%)	NA
Unreported*	5 (3%)	NA
Triggering events		
Viral, bacterial and/or other unknown infection	101 (80%)	NA
Physical and emotional trauma and others	25 (20%)	NA
Diagnosed with any autoimmune diseases		
Yes	50 (30%)	8 (10%)
No	110 (66%)	69 (83%)
Unreported*	6 (4%)	6 (7%)
Diagnosed with any cancer		
Yes	21 (13%)	2 (2%)
No	137 (82%)	71 (86%)
Unreported*	8 (5%)	10 (12%)
Diagnosed with kidney disease		
Yes	5 (3%)	0 (0%)
No	154 (93%)	73 (88%)
Unreported*	7 (4%)	10 (12%)

*Subjects did not provide information and were assigned to either CFS or normal based on the enrollment link they chose for registering for the study

Onset of CFS symptoms was reported by the participants. Duration of the CFS disease was calculated and reported (Table 2): 17% of the ME/CFS participants reported to have CFS between 1 and 5 years, 18% between 6 and 10 years, 13% between 11 and 15 years, 12% between 16 and 20 years, 21% between 21 and 30 years, 16% between 31 and 63 years, and 2% did not report.

Approximately half (49%) of the ME/CFS patients reported being house-bound or bedridden due to the symptoms of the disease (Table 2). In this study, we considered house-bound or bedridden patients as having severe symptoms, and the rest as having mild to moderate symptoms [7].

Seventy-six percent (76%) of the ME/CFS patients reported a triggering event before the onset of the disease. Among them, eighty percent (80%) reported an assignable viral or bacterial infection as the trigger, while twenty percent (20%) reported other physical and/or emotional traumatic events as the trigger (Table 2). Among the viral and bacterial infections, organisms listed in the questionnaire included: EBV, Mononucleosis, Flu, H. Pylori, Pneumonia, Strep Throat and COVID (Table 2). In addition, thirty percent (30%) of the ME/CFS patients have or have had autoimmune diseases (e.g. Hashimoto's thyroiditis, Fibromyalgia, IBD, POTS, Grave’s Disease, Crohn’s disease, Psoriasis, Rheumatoid arthritis and Sjogren's syndrome). Thirteen percent (13%) have or have had various cancers. Five percent (5%) have or have had kidney diseases (Table 2). Eighty-five percent (85%) of the CFS patients were taking medications (currently or within the last 4 weeks), including Antibiotics, Antihistamines, Immunomodulatory, and Non-Steroidal Anti-Inflammatory drugs, comparing to 36% of the normal controls listing similar medications (not listed).

### AIP results reveal genes that are up regulated in bedridden CFS patients

MCD blood samples were collected from 163 CFS and 79 control participants at the first timepoint, and samples were also collected from 33 CFS and 15 control participants at the second timepoint (~ 19% of each group). The second timepoint samples were collected voluntarily approximately 4 weeks after the first time point. The purpose for testing the second time point was to verify there was no sampling bias between the collections. Together, the AIP testing was completed for a total of 287 MCD samples (out of 240 participants, including first and second timepoint collections). Out of the 287 tests, 267 tests (93.0%) passed QC acceptance.

The testing was carried out in 2 distinct batches. To assess potential batch effects, both batches were combined and principal component analysis (PCA) was performed to visualize clusters of normalization genes and immune module genes separately. Replicate samples displayed a high Spearman’s rank correlation coefficient (r = 0.86, p < 2.2e−16), but multivariable regression models revealed a downward shift in mean unique to timepoint. COMBAT [24] empirical bayes standardization resolved this deviation, suggesting this relatively small batch effect was technical in nature.

**Fig. 3:**
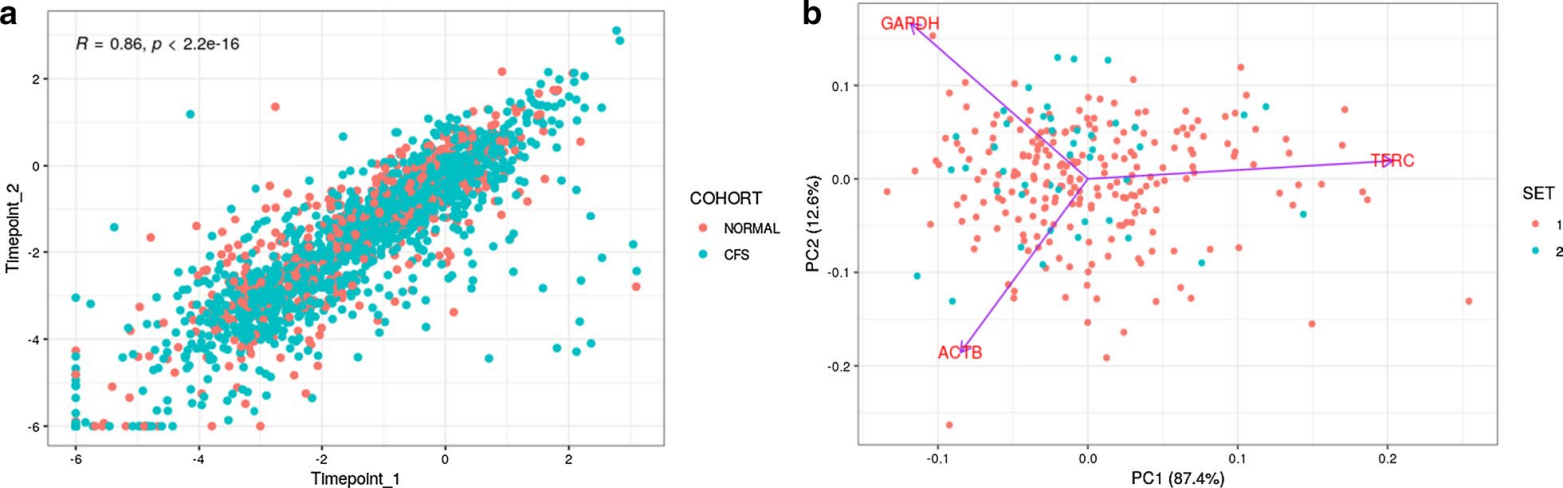
Correlation of gene expression measurements between samples collected at two timepoints (data points combining all 51 genes per sample for all samples)(**a**); Correlation of gene expression measurements of house-keeping genes between two batches (**b**). Wilcoxon signed-rank tests were performed between replicates on both the raw and normalized relative florescent units (RFU) of three housekeeping genes (ACTB, GAPDH, TFRC). To assess potential batch effects, both batches were combined and principal component analysis (PCA) was performed to visualize clusters of normalization genes and immune module genes separately

When examining concordance between the samples collected at two timepoints, the scatter plot in Fig. 3a showed a good correlation (r = 0.86) between the timepoint 1 and 2 samples. Each gene signal (raw RFU) is normalized against the geometric mean of the 3 house-keeping genes (ACTB, GAPDH, TFRC). When examining the 3 house-keeping genes between the 2 batch data sets, the PCA plot showed no observable difference in house-keeping genes by the 2 data sets, therefore there is no batch effect in sample testing (Fig. 3b). The AIP results in the subsequent analyses were based on the first time point (excluding repetitive samples from the same participants), in which all samples were collected from unique participants of either the CFS patients or the normal controls.

**Fig. 4:**
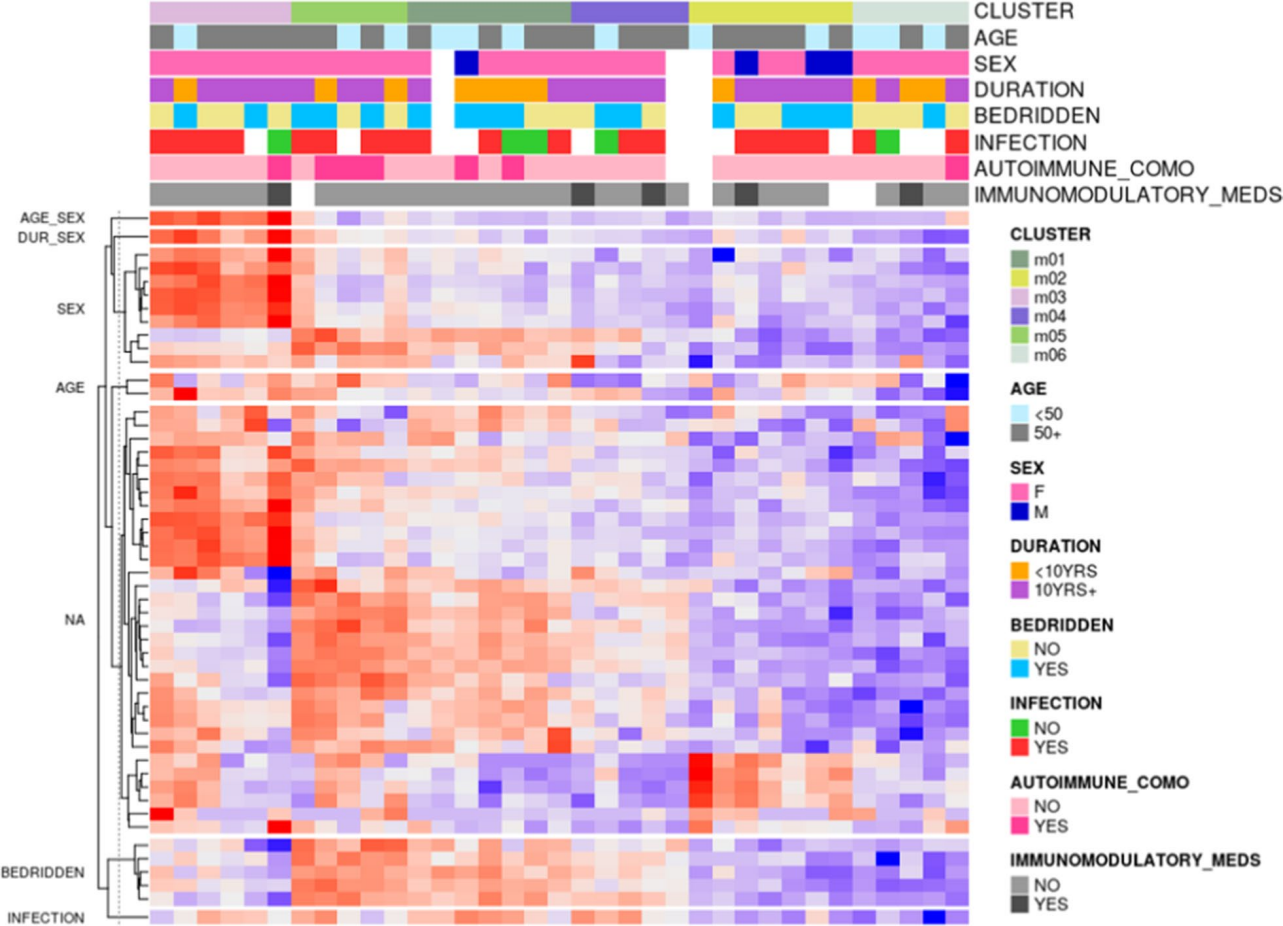
Unsupervised Clustering Analysis (heat map) showing the relationship between genes significantly up- or down-regulated in participant subsets with certain demographic or clinical attributes. Single columns represent patients and column annotations align to their associated clinical or demographic categories on the top. Individual rows underneath denote the 51 AIP panel genes split by differentially expressed subgroups for that gene on the left portion of the heatmap

From the CFS cohort, unsupervised consensus clustering was conducted to reveal a heat map showing the relationship between genes significantly up- or down-regulated in patient subsets with certain demographic or clinical attributes, such as age, gender, disease duration, prior viral infection, bedridden status, autoimmune comorbidity, and immunomodulatory medicine (Fig. 4). Six major clusters were unveiled after merging several candidate clusters (labeled as m01–m06 in the heatmap). These groupings confirm categorical gene expression modulation also reported from prior regression testing, such as enrichment in IFIT1, ISG15, and IFI6 in female patients and higher expression of APOL6 and SLC2A3 in patients 55 years and older. In addition, there were also visible expression differences which may be explained by unreported factors from patients.

**Fig. 5:**
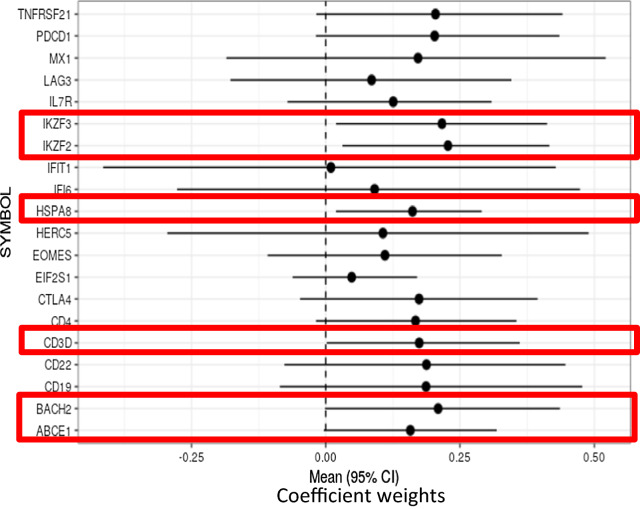
Univariable bootstrapped analysis identified 6 genes of interest (red box) differentially expressed by CFS bedridden Status. The forest plot visually displays the mean coefficient values and 95% lower and upper confidence interval boundaries for each gene and value listed in Table 3

Next, we analyzed genes that were differentially expressed between two subsets of the CFS patients: self-reported bedridden patients (n = 72) and self-reported non-bedridden patients (n = 72). The bedridden patients were considered to have severe disease. Regression bootstrapping was performed on the genes which were borderline modulated between CFS bedridden and CFS non-bedridden participants per linear models controlling for demographic and clinical confounders such as age, sex, prior infection, and symptom duration. This analysis revealed six genes with significant (p < 0.05) differential expression by bedridden status: IKZF2, IKZF3, ABCE1, BACH2, CD3D and HSPA8 (Table 3). A forest plot showed the coefficient means and 95% confidence intervals for each gene in Fig. 5. A bootstrapped multivariable model on bedridden status was performed controlling for age and sex. The genes most associated with bedridden status when controlled for age and sex were HSPA8, IKZF2, and IKZF3 (data not shown).

**Table 3:**
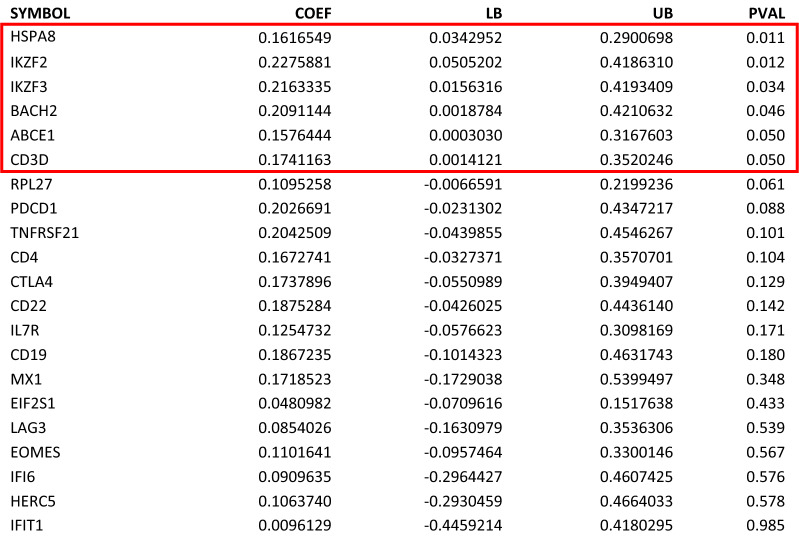
Univariable bootstrapped analysis Identified 6 genes differentiated by bedridden status in CFS patients (p < 0.05)

Regression models from 21 genes which were borderline modulated between CFS bedridden and CFS non-bedridden patients underwent residual resampling to account for sample bias and potential noise in the dataset. The bootstrapped confidence interval p-value (CFS bedridden vs. CFS non-bedridden). The gene symbol, coefficient means, lower and upper bound 95% confidence interval values, and confidence interval p-values are displayed

### AIP results reveal genes that are upregulated in CFS severe cases with autoimmune comorbidity

**Fig. 6:**
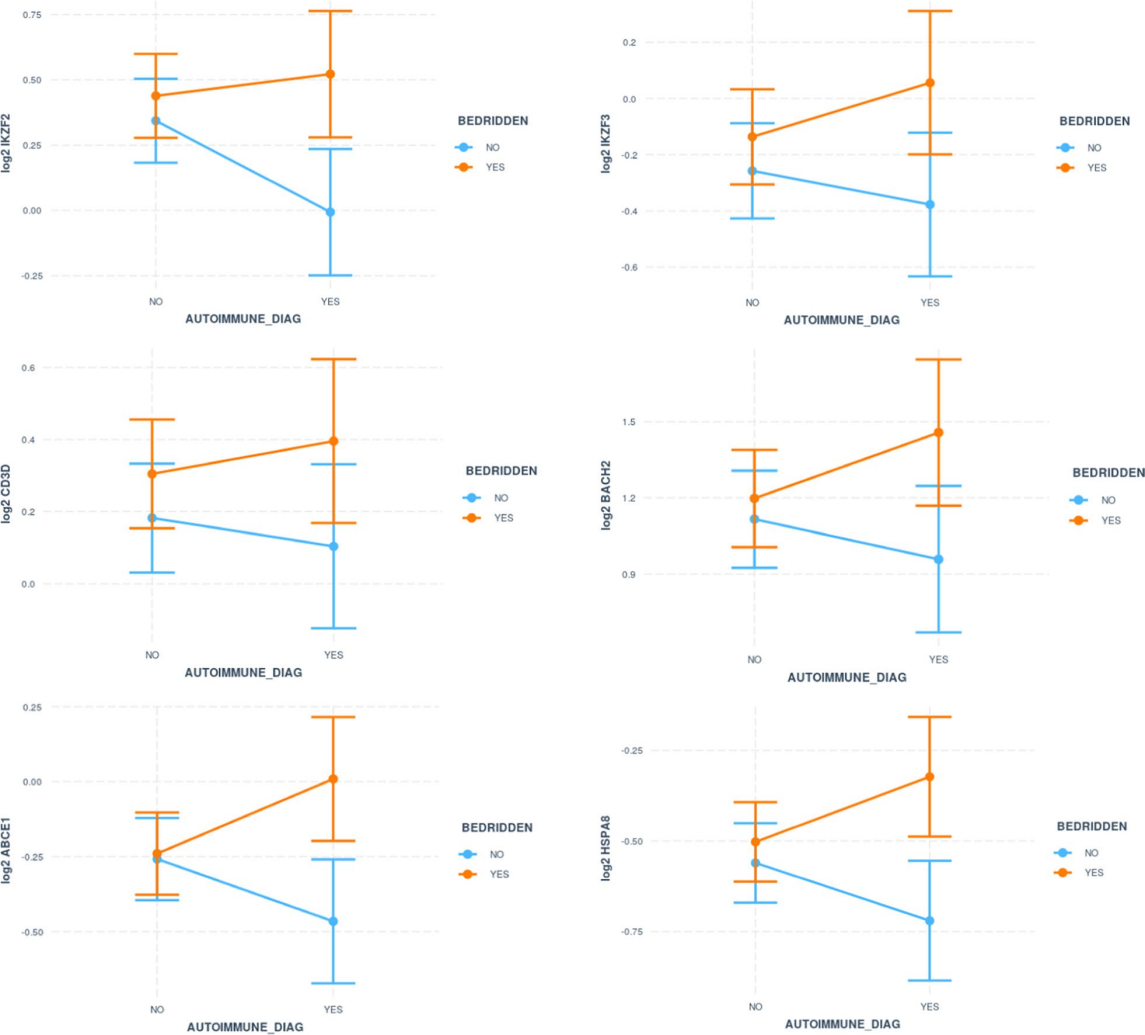
Bedridden CFS patients with other autoimmune diseases display larger differential expression in the same six genes (IKZF2, IKZF3, ABCE1, BACH2, CD3D and HSPA8)

A subset of CFS patients also had an autoimmune etiology [16]. In this study, 30% of CFS participants reported having one or more autoimmune diseases that co-exist with CFS. For these 44 patients, we compared gene expression levels of the 6 genes between the 22 bedridden and the 22 non-bedridden patients. Each of the 6 genes exhibited a greater separation in this subset of CFS patients with other autoimmune diseases (Fig. 6), suggesting an additive effect of fatigue related gene expression in CFS and other autoimmune etiologies.

### Viral testing yields few detectable viruses

The MCD blood samples were also used to extract DNA which was tested for several viral infections using a real time PCR method at Coppe Laboratories, including HHV-6A and HHV-6B, HHV-7, EBV and CMV. Among the 240 participants tested with the viral assays, 9 samples showed positive results (Table 4). One CFS sample was positive for EBV, and 8 were positive for HHV6 (including 7 CFS samples and 1 normal). The p-value between the CFS and control groups was p = 0.074. Among the HHV6 positives, further analysis showed 4 of them were with the HHV6B genotype, and 1 of them was with the HHV6A genotype.

**Table 4:** Viral detection tests yield few detectable viruses indicative of active ongoing infection

MCD ID	Virus dtected	HHV6A/6B subtyping	Ct Value	Copies/mL	Internal control RPP30 (Ct)	Cohort
100036628	HHV6 Detected	HHV6A Detected	24.29	229,568.9	22.5	Normal
100036589	HHV6 Detected	N/A	37.21	47.3	23.63	CFS
100037181	HHV6 Detected	N/A	38.84	16.2	21.12	CFS
100036608	HHV6 Detected	HHV6B Detected	24.72	173,073.7	22.68	CFS
100037202	HHV6 Detected	HHV6B Detected	23.94	288,915.2	21.79	CFS
100037159	HHV6 Detected	HHV6B Detected	24.5	199,985.5	22.54	CFS
100036858	HHV6 Detected	HHV6B Detected	23.07	511,666.7	20.99	CFS
100036599	HHV6 Detected	N/A	39.38	11.4	21.08	CFS
100037041	EBV Detected	N/A	37.67	6.7	24.86	CFS

## Discussion

### Patient centric approach

In this study, we took a social media-based patient centric recruitment and at-home sample collection approach. Direct-to-participant (D2P) recruitment and remote collection of samples allowed potential enrollment of a broader study population than was not easily achievable through a conventional site-based model. Patient advocates, such as social influencers, were contacted to share resources and aid in the recruitment of participants. Advocates and influencers were also invited to enroll and participate in the study to better understand the study experience and provide context for their shareable content. D2P participants interacted with the study through a study-specific app that was available for both iOS and Android operating systems. The app provided an end-to-end solution for deploying, completing, and managing the D2P study activities.

The D2P approach was advantageous in that it enabled participants to collect samples from geographically diverse locations in the US without the need to set up multiple clinical sites. Participants did not need to visit a physician’s office and were able to self-collect samples at home. This was particularly convenient for CFS patients with severe symptoms who are house- or bed-bound. The MCD fingerstick whole blood collection device allows self-collection of small amounts of blood. The participants were able to simultaneously collect and ship blood and urine samples needed for conducting multiple tests. This approach enabled the study to be conducted at a relatively low cost when compared to the anticipated costs for a site-based approach and allowed faster turn-around time. Some limitations of the approach include: 1) Self-reported patient medical history is not verified. 2) Discarded/missing samples due to improper collection procedure by the participants could lead to loss of samples. In this study, sample attrition rate was 3% for MCD blood samples and 8% for urine samples, which were either discarded or not received. The success rate for sample collection was 92% for urine and 97% for MCD.

### CFS and T cell and B cell functions

CFS is a complex clinical condition of unknown etiology, characterized by persistent or intermittent fatigue that is not the result of recent exertion and does not improve with rest, resulting in a significant reduction in the patient's previous normal activity. CFS is a multi-system disease, with altered immune, musculoskeletal, endocrine, neurological and cardiovascular systems. In this study, we have identified 6 genes using a molecular profiling approach. These genes (IKZF2, IKZF3, ABCE1, BACH2, CD3D and HSPA8) have been implicated in various biological functions, particularly in T cell and B cell biology.

The Ikaros zinc-finger family transcription factors (IKZF TFs) are important regulators of lymphocyte development and differentiation and are also highly expressed in B cell malignancies and are required for cancer cell growth and survival [25–27]. Moreover, IKZF TFs negatively control the functional properties of many immune cells. Specifically, IKZF3 plays an essential role in regulation of B-cell differentiation, proliferation, and maturation to an effector state. It is involved in regulating BCL2 expression and controlling apoptosis in T-cells in an IL2-dependent manner. Diseases associated with IKZF3 include Immunodeficiency 84 and Chronic Lymphocytic Leukemia. CD3D is a T-cell receptor/CD3 complex and is involved in T-cell development and signal transduction. IKZF2, another member of the Ikaros TFs, controls lymphocyte development, promotes quiescence, maintains the inhibitory function of regulatory T cells, and is frequently deleted in hypodiploid B-acute lymphoblastic leukemias (B-ALLs) [28]. BACH2 is a basic leucine zipper transcription factor expressed in B cells from the pro-B cells to mature B cells and is downregulated during the maturation to plasma cells. BACH2 is involved in primary adaptive immune response involving T cells and B cells and enables sequence-specific double-stranded DNA binding activity [29]. BACH2 is essential for the differentiation of stem-like CD8+ T cells during chronic viral infection. Overexpression of BACH2 upregulates IKZF2 gene [30].

IKZF3 (Aiolos) and IKZF2 (Helios), and BACH2 have been implicated in other autoimmune disease such as systemic lupus erythematosus (SLE) and Rheumatoid Arthritis (RA) [25, 31–36]. In this study, the expression of these genes was increased in severe cases of CFS and greater upregulation was observed in those who suffer from comorbid autoimmune diseases, suggesting that CFS is, in part, an autoimmune disease. These genes can be utilized as potential biomarkers to distinguish mild CFS from severe CFS with autoimmune comorbidity. Furthermore, accumulating evidence demonstrated that downregulation of IKZF3 (Aiolos) and IKZF1 (Ikaros), two members of the IKZF family, in malignant plasma cells as well as in adaptative and innate lymphocytes is key for the anti-myeloma activity of Immunomodulatory drugs (IMiDs) [37]. In addition, IKZF1, IKZF3 and IKZF2 (Helios) have been implicated in SLE pathogenesis. There is strong evidence that therapeutic targeting of Ikaros and Aiolos can ameliorate key pathogenic processes in human SLE [37].

### CFS, viral infection and cancer

CFS is characterized by fatigue and severe disability. Besides fatigue, certain aspects of immune dysfunction appears to be present in both CFS and cancer [38]. The underlying cause of CFS is unknown due to its heterogeneity, but in many cases, it is thought to be triggered by an abnormal immune response to an agent, such as a viral infection, that results in chronic immune activation. The immunologic changes in CFS and its possible relationship with infection have prompted investigators to consider whether CFS could also be associated with an elevated risk of cancer. A 2013 study has reported that CFS was present in 0.5% of cancer cases among US elderly and 0.5% of controls [39]. CFS was associated with an increased risk of non-Hodgkin’s lymphoma (NHL) and specifically NHL subtypes diffuse large B cell lymphoma, marginal zone lymphoma, and B-cell NHL not otherwise specified (NOS) [39]. In a recent clinic-based study, with a carefully selected matched cohort of 59 confirmed CFS and 54 control cases, the authors have identified statistically significant increased risks of autoimmune disease and cancer among the first-degree relatives of ME/CFS cases [17].

IKZF2 is highly expressed in leukemic stem cells (LSCs) and its deficiency results in defective LSC function. IKZF2 has been shown to drive leukemia stem cell self-renewal and inhibits myeloid differentiation. Regulation of the AML LSC program by IKZF2 thus provides a rationale to therapeutically target IKZF2 in myeloid leukemia [28].

Abnormalities in ribonuclease (RNase) L and hyperactivation of nuclear factor kappa beta (NF-κB) are present in CFS and in prostate cancer [40]. One of the key antiviral effectors is the IFN-inducible oligoadenylate synthetase/ribonuclease L (OAS/RNase L) pathway, which is activated by double-stranded RNA to synthesize unique oligoadenylates, 2-5A, to activate RNase L. RNase L exerts an antiviral effect by cleaving diverse RNA substrates, therefore limiting viral replication; many viruses have evolved mechanisms to counteract the OAS/RNase L pathway [40]. ATP-binding cassette E1 (ABCE1) transporter, identified as an inhibitor of RNase L, regulates RNase L activity and RNase L-induced autophagy during viral infections [40]. In this study, we identified ABCE1 as one of the six genes upregulated in severe CFS cases. This suggests the RNase L pathway may be impaired in these individuals due to elevated ABCE1 expression.

HSPA8 is implicated in a signal transduction pathway in the abnormal proliferation of chronic myeloid leukemia (CML) cells, suggesting that the chaperone HSPA8 and CCND1 contribute to the abnormal behavior of CML cells and represent an interesting target for new therapies [41].

Clinical activity from B-cell depletion using anti-CD20 antibody rituximab has been utilized in treating CFS patients [42]. However, improvement of fatigue was observed in the responders 3 to 7 months after the treatment and the initial rapid B-cell depletion. This data suggested that CFS may be an autoimmune disease, and the delayed response to rituximab could be due to the elimination of the disease-associated autoantibodies. In a separate study, three patients, with 7–10 years of CFS disease duration, had substantial relief of all symptoms related to CFS after rituximab intervention. Overall, there was a major recovery of all symptoms lasting until 40 weeks after treatment, followed by a gradual returning of symptoms. CFS may be amenable to therapeutic interventions aimed at modifying B-cell number and function [43]. It is also speculated that this response pattern could be related to interaction of B-cell and T-cell in antigen presentation. Our finding of up-regulation of both B-cell and T-cell associated genes support this hypothesis.

In this study, we investigated the expression of genes specific to key immune functions in ME/CFS patients. The six genes we identified are strongly related to T cell and B cell functions. The results could hopefully shed some light in further exploring the biological connections between CFS, autoimmune disease and cancer, with an aim of identifying possible therapeutics of the disease. Research focusing on etiology and pathogenesis of CFS could help unravel biological roots of the disease. Additionally, benefits of discriminating differentiated symptoms of CFS from autoimmune and cancer could prove valuable in searching for immunity and oncogenic related therapeutics.

Before the pandemic, estimated 1.5–3 million Americans were suffering from CFS in US alone. An unprecedented wave of CFS-like illness related to COVID infection will appear over the next few years, with profound societal costs. The striking similarities between CFS and long COVID suggest commonality in the disease mechanisms may exist. Studies on the mechanism and treatment of CFS will facilitate research on the long COVID.

## Conclusions

The significance of our study is two-fold: (1) gene expression biomarkers may be used in identifying or differentiating subsets of ME/CFS patients having different levels of disease severity. These gene targets may also represent opportunities for new therapeutic modalities for the treatment of ME/CFS. (2) The use of social media engaged patient recruitment and at-home sample collection represents a novel approach for conducting clinical research which saves cost, time and eliminates travel for office visits.

## Data Availability

Yes.

## Abbreviations

ME/CFS: Myalgic Encephalomyelitis/Chronic Fatigue Syndrome
CFS: Chronic Fatigue Syndrome
IMiDs: Immunomodulatory drugs
RA: Rheumatoid Arthritis
ePRO: Electronic patient reported outcome
IRB: Institutional Review Board
MCD: Micro Collection Device
EBV (or HHV4): Epstein-Barr Virus
CMV (or HHV5): Cytomegalovirus
HHV6: Human herpesvirus 6
HHV7: Human herpesvirus 7
IKZF TFs: Ikaros zinc-finger family transcription factors
SLE: Systemic lupus erythematosus
D2P: Direct-to-participant
RFU: Relative florescent units
PCA: Principal component analysis
RSEC: Resampling-based sequential ensemble clustering
CML: Chronic myeloid leukemia
IOM: Institute of Medicine
